# Supplementary material for: Reverse-Correlation Analysis of the Mechanosensation Circuit and Behavior in C. elegans Reveals Temporal and Spatial Encoding
Source: Sci Rep. 2019 Mar 26;9:5182. doi: 10.1038/s41598-019-41349-0 (PMC6435754; doi:10.1038/s41598-019-41349-0)
Supplement: Supplementary file 1 — SI [file 41598_2019_41349_MOESM1_ESM.pdf]

Reverse-Correlation Analysis of the Mechanosensation Circuit and Behavior in *C. elegans* Reveals Temporal and Spatial Encoding

Daniel A. Porto[1], John Giblin[2], Yiran Zhao[2], Hang Lu[1,2,3*]

[1]Interdisciplinary Bioengineering Program, Georgia Institute of Technology, USA

[2]Department of Biomedical Engineering, Georgia Institute of Technology, USA

[3]School of Chemical & Biomolecular Engineering, Georgia Institute of Technology, USA

*Correspondence should be addressed to HL: hang.lu@gatech.edu, 1-404-894-8473

Movie S1: Example trial of white noise stimulation in our platform. An m-sequence light signal is delivered to either the anterior or posterior segment of the animal while simultaneously being tracked. For each trial, various discrete and continuous behaviors are quantified (Methods).

Movie S2: Sample filter computed using BWA as a function of sample size used for the computation.

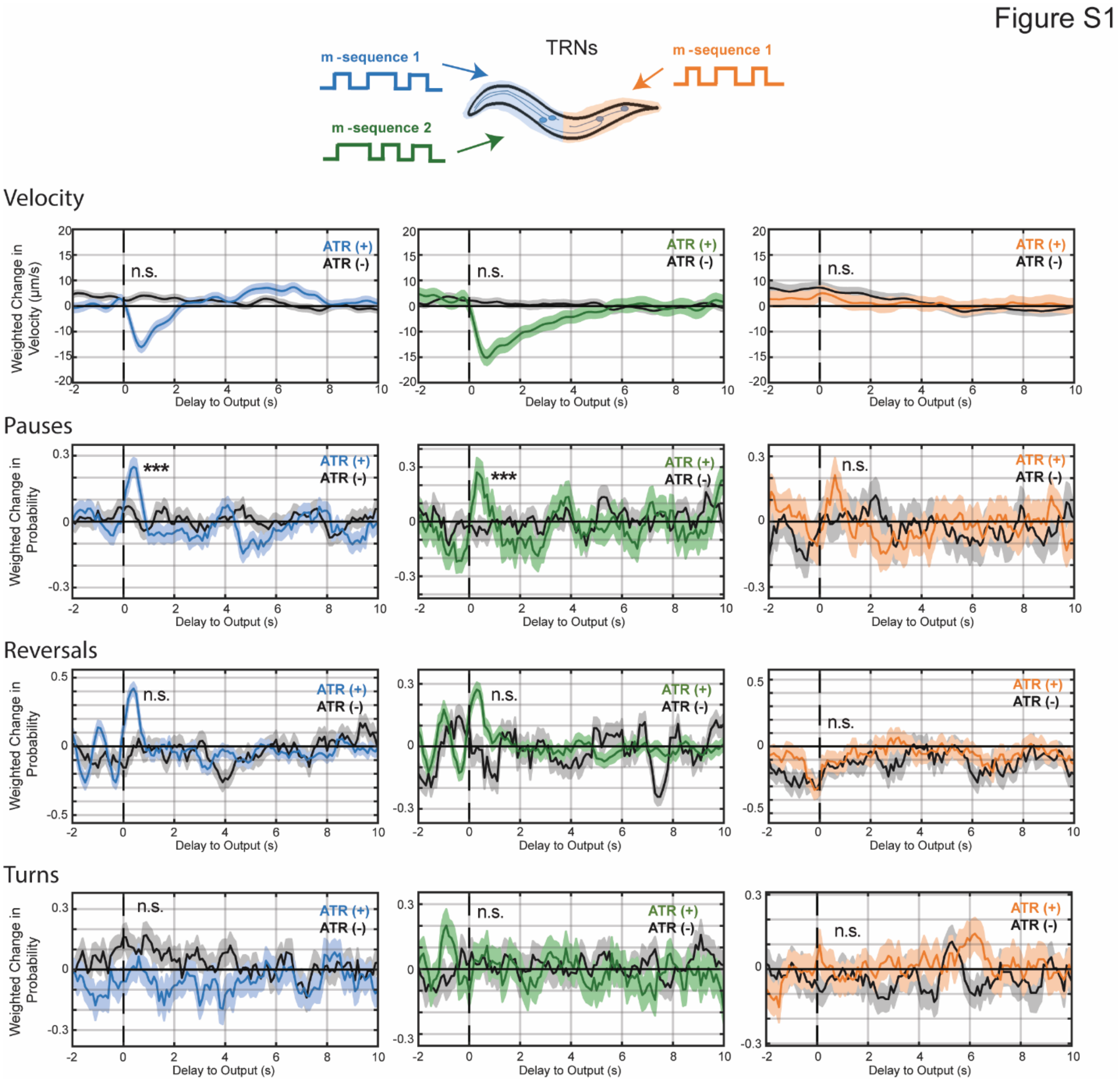

Figure S1: Additional linear filters for TRNs. Linear filters computed for various behaviors when stimulating the anterior TRNs with an m-sequence signal (left), a different m-sequence signal (center), and the posterior TRNs (right). Dark line and light shade represent BWA and SEM, respectively. Colored plots represent filters computed from ATR-fed animals, black plots represent filters computed from control (not ATR-fed) animals. Statistical significance of filters determined by comparison with shuffled data (Fig S5-S6, ***Top 1%, and not significant otherwise). Sample sizes listed in Table S1.

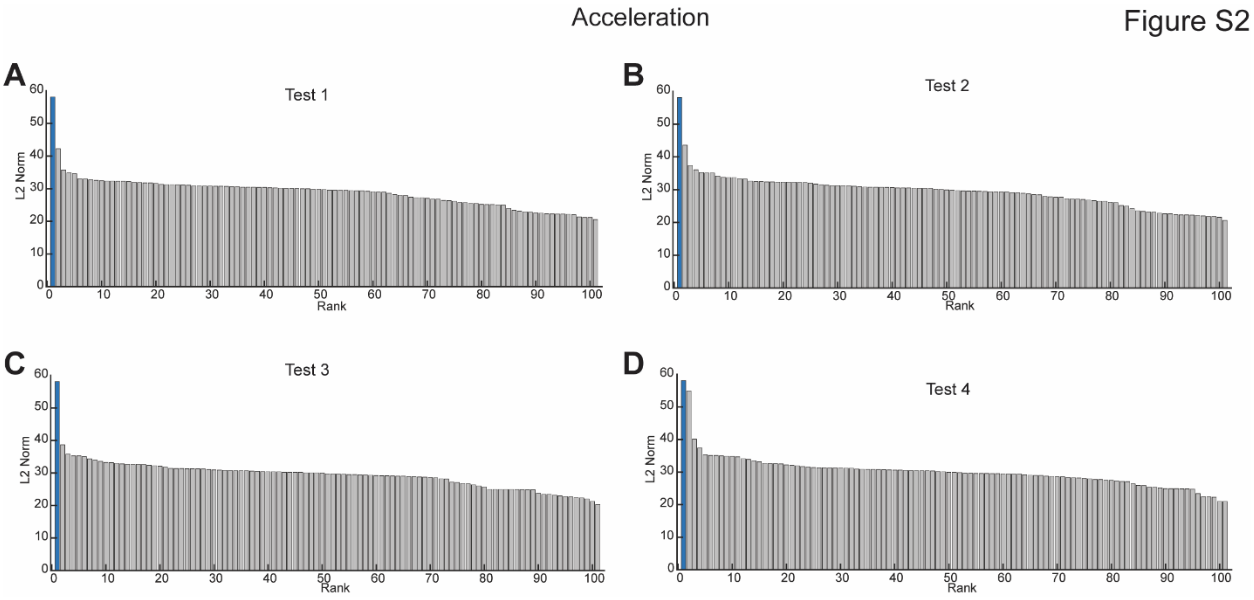

Figure S2: Comparison of shuffled data significance tests. Results from comparison of four methods of shuffling data for statistical significance tests of linear filters. (A) Cyclic shuffling of stimulus vector by a random integer. (B) Cyclic shuffling of behavior vector by a random integer. (C) Random permutation of stimulus vector. (D) Random permutation of behavior vector. Bar plots represent the magnitude of filters, computed as the L2 norm, and are plotted in ranked order from highest to lowest magnitude. Colored bar represents appropriately computed filter, gray bars represent filters computers with shuffled data.

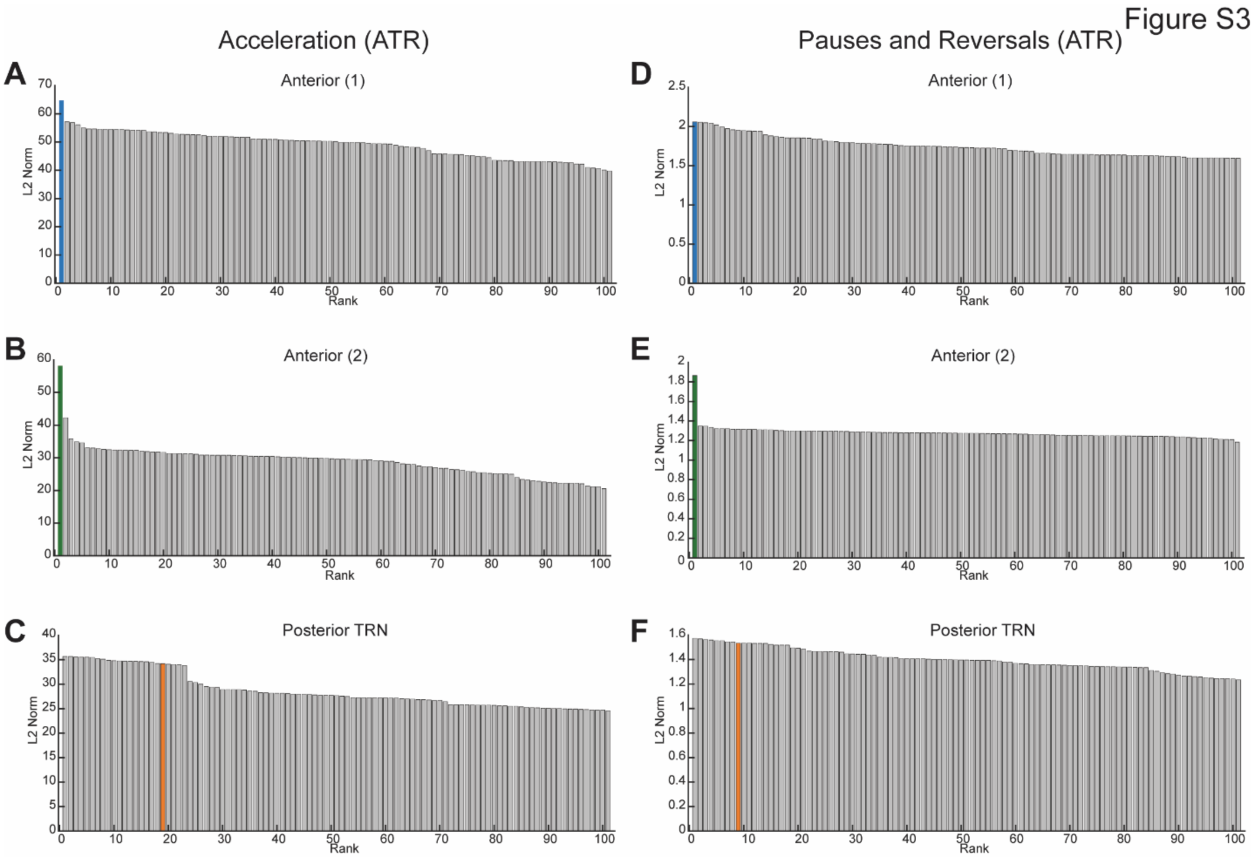

Figure S3: Significance test results for linear filters for TRNs. Results from shuffled data significance tests for linear filters computed for activation of TRNs in ATR-fed animals in Figure 2. (A-C) Significance test results for computed filters for acceleration for anterior TRNs (A,B) and posterior TRNs (C). (D-F) Significance test results for computed filters for pauses and reversals for anterior TRNs (D,E) and posterior TRNs (F). Bar plots represent the magnitude of filters, computed as the L2 norm, and are plotted in ranked order from highest to lowest magnitude. Colored bar represents appropriately computed filter, gray bars represent filters computers with shuffled data.

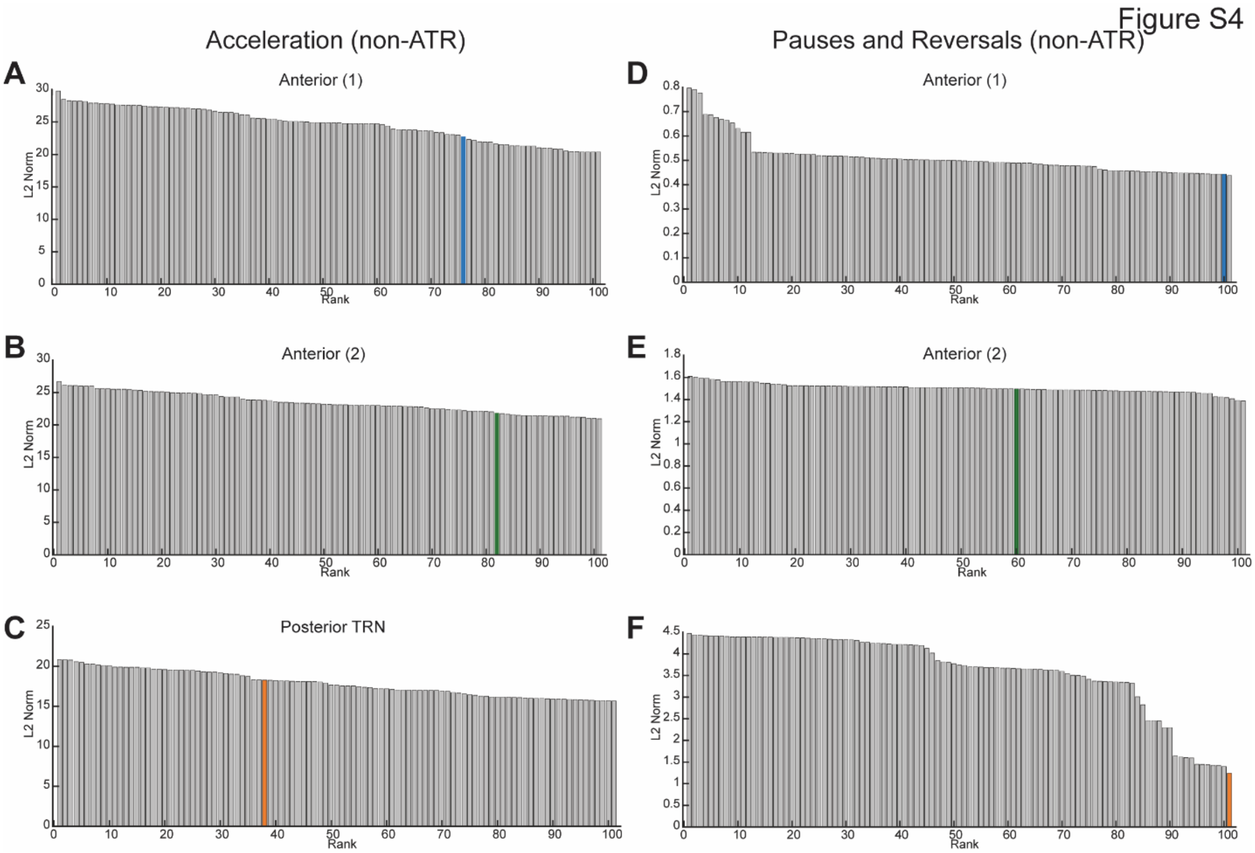

Figure S4: Significance test results of nonATR control data for linear filters for TRNs. Results from shuffled data significance tests for linear filters computed for activation of TRNs in control, or nonATR-fed animals, in Figure 2. (A-C) Significance test results for computed filters for acceleration for anterior TRNs (A,B) and posterior TRNs (C). (D-F) Significance test results for computed filters for pauses and reversals for anterior TRNs (D,E) and posterior TRNs (F). Bar plots represent the magnitude of filters, computed as the L2 norm, and are plotted in ranked order from highest to lowest magnitude. Colored bar represents appropriately computed filter, gray bars represent filters computers with shuffled data.

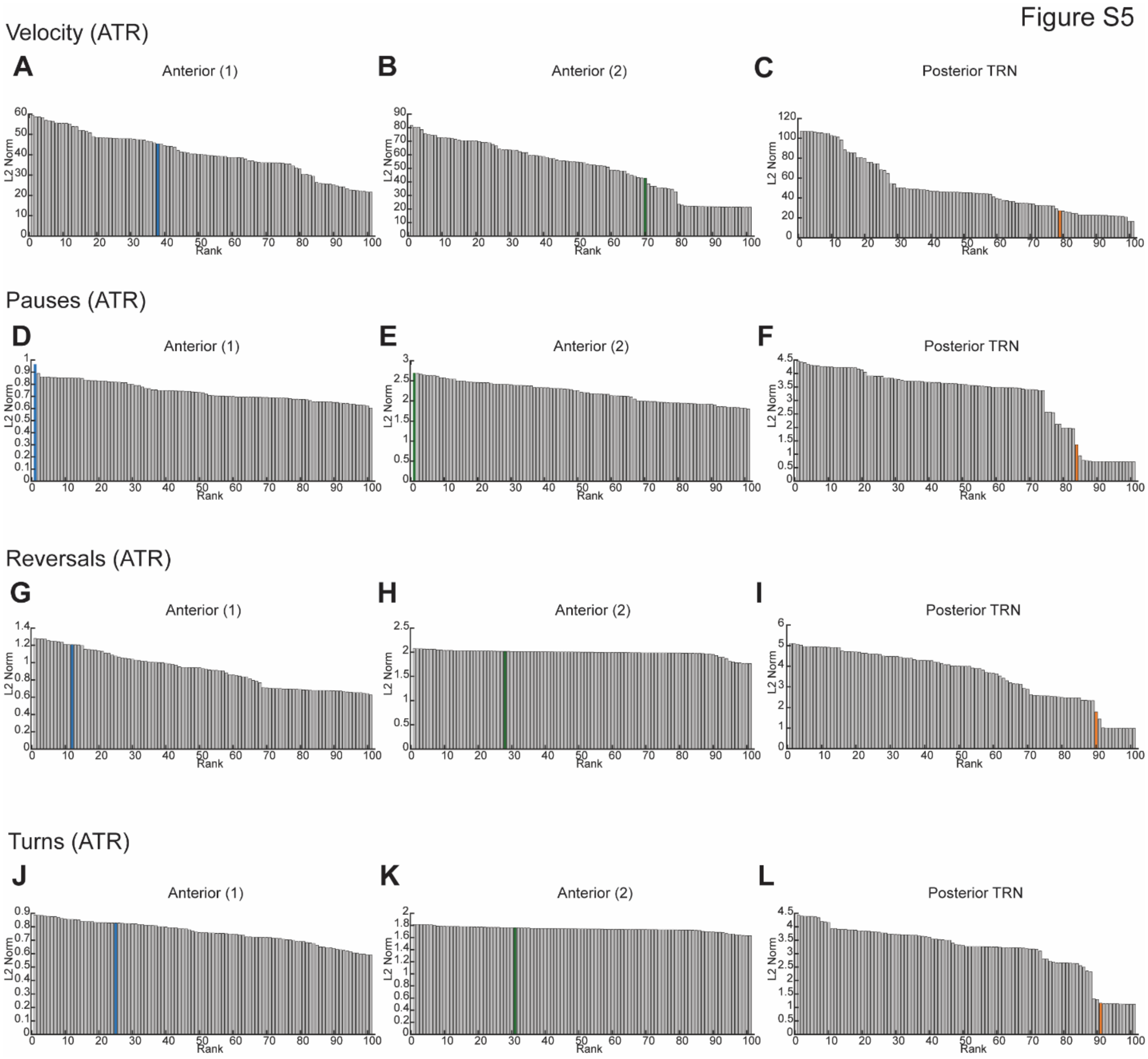

Figure S5: Significance test results for additional linear filters for TRNs. Results from shuffled data significance tests for linear filters computed for activation of TRNs in ATR-fed animals in Figure S1. Significance test results for computed filters for velocity (A-C), pauses (D-F), reversals (G-I), and Turns (J-L) for anterior TRNs and posterior TRNs. Bar plots represent the magnitude of filters, computed as the L2 norm, and are plotted in ranked order from highest to lowest magnitude. Colored bar represents appropriately computed filter, gray bars represent filters computers with shuffled data.

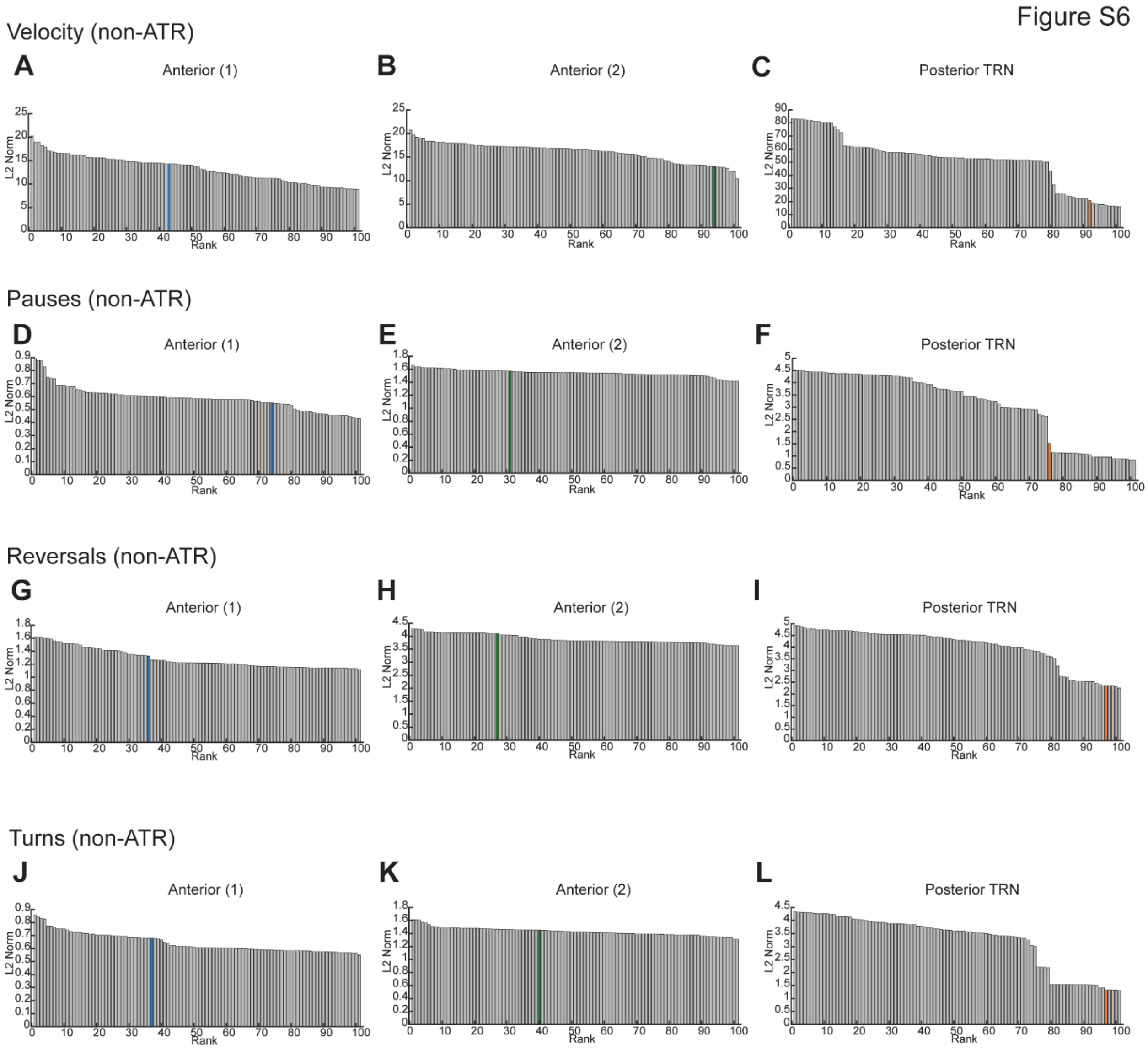

Figure S6: Significance test results of nonATR control data for additional linear filters for TRNs. Results from shuffled data significance tests for linear filters computed for activation of TRNs in control, or nonATR-fed animals, in Figure S1. Significance test results for computed filters for velocity (A-C), pauses (D-F), reversals (G-I), and Turns (J-L) for anterior TRNs and posterior TRNs. Bar plots represent the magnitude of filters, computed as the L2 norm, and are plotted in ranked order from highest to lowest magnitude. Colored bar represents appropriately computed filter, gray bars represent filters computers with shuffled data.

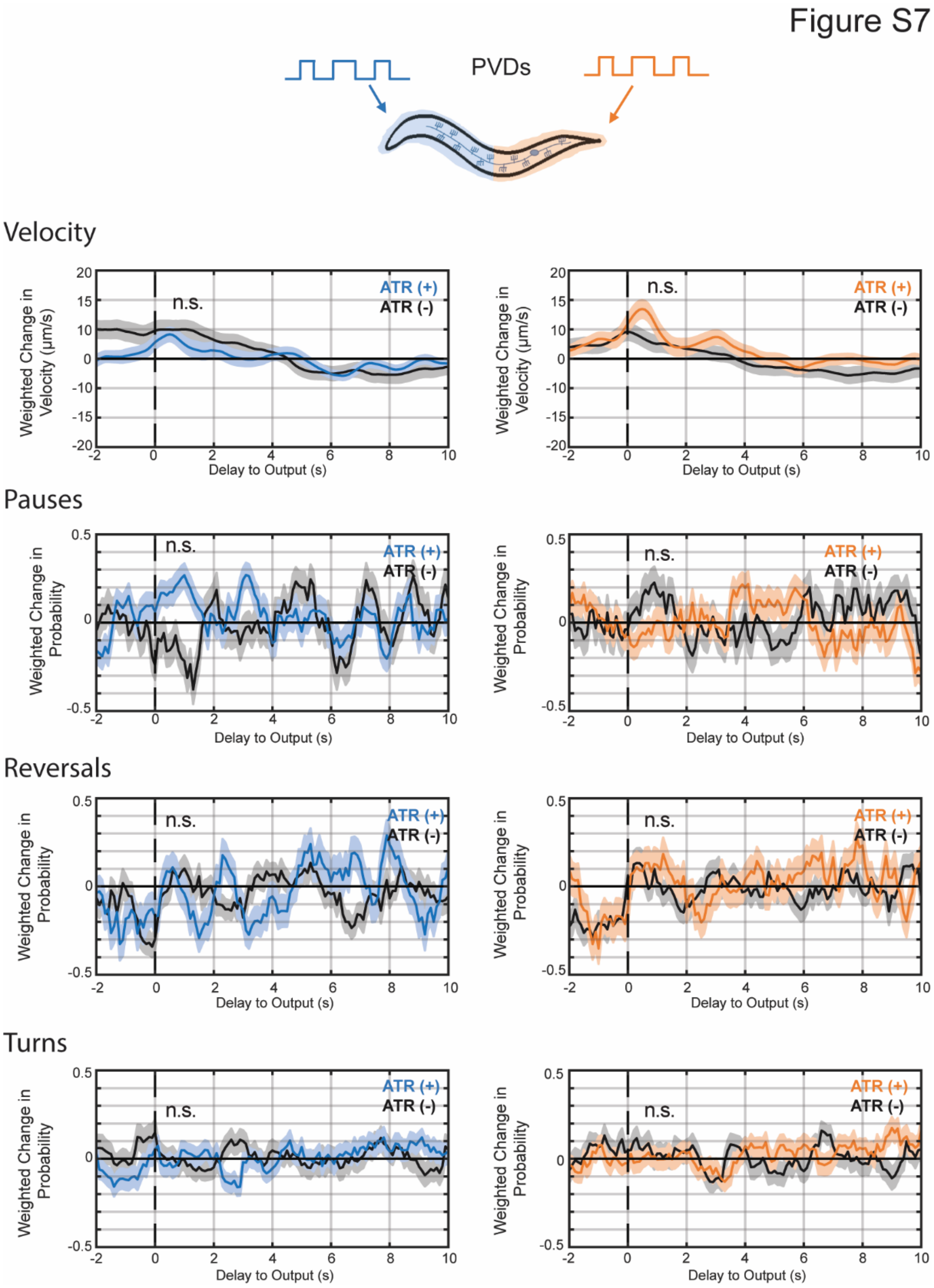

Figure S7: Additional linear filters for PVDs. Linear filters computed for various behaviors when stimulating the anterior (left) and posterior (right) PVDs with an m-sequence signal. Dark line and light shade represent BWA and SEM, respectively. Colored plots represent filters computed from ATR-fed animals, black plots represent filters computed from control (not ATR-fed) animals. Statistical significance of filters determined by comparison with shuffled data (Fig S10-S11, ns not significant). Sample sizes are listed Table S1.

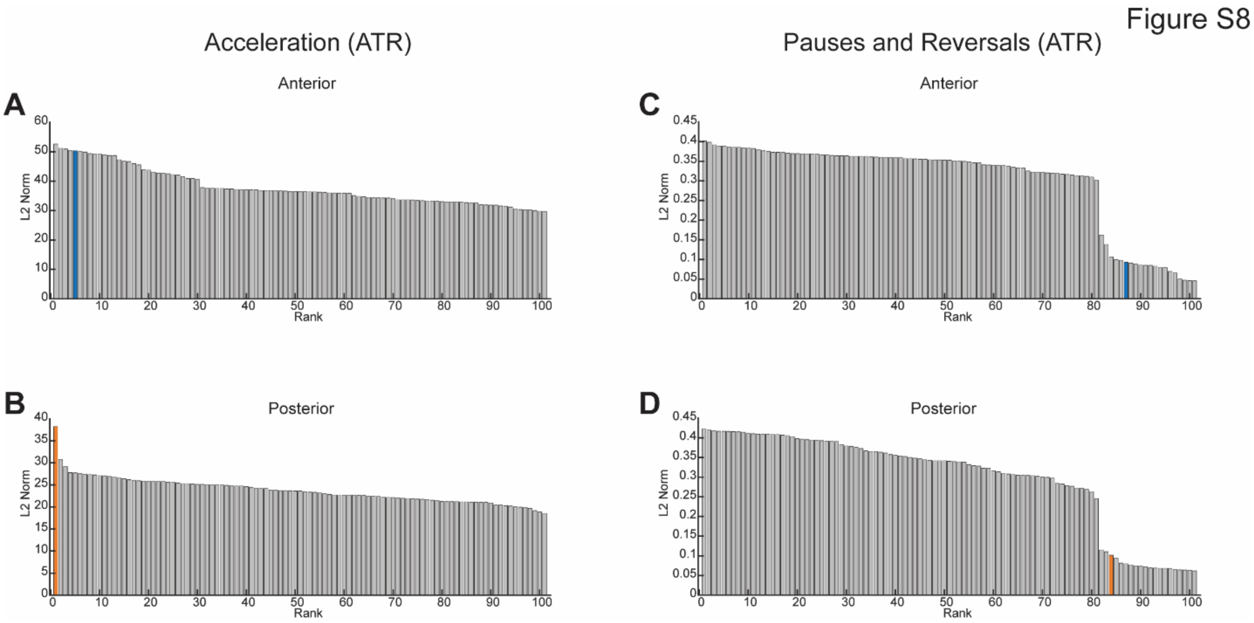

Figure S8: Significance test results for linear filters for PVD. Results from shuffled data significance tests for linear filters computed for activation of PVD in ATR-fed animals in Figure 3. (A,B) Significance test results for computed filters for acceleration for anterior (A) and posterior (B) segments of PVD. (C,D) Significance test results for computed filters for pauses and reversals for anterior (C) and posterior (D) segments of PVD. Bar plots represent the magnitude of filters, computed as the L2 norm, and are plotted in ranked order from highest to lowest magnitude. Colored bar represents appropriately computed filter, gray bars represent filters computers with shuffled data.

Figure S9

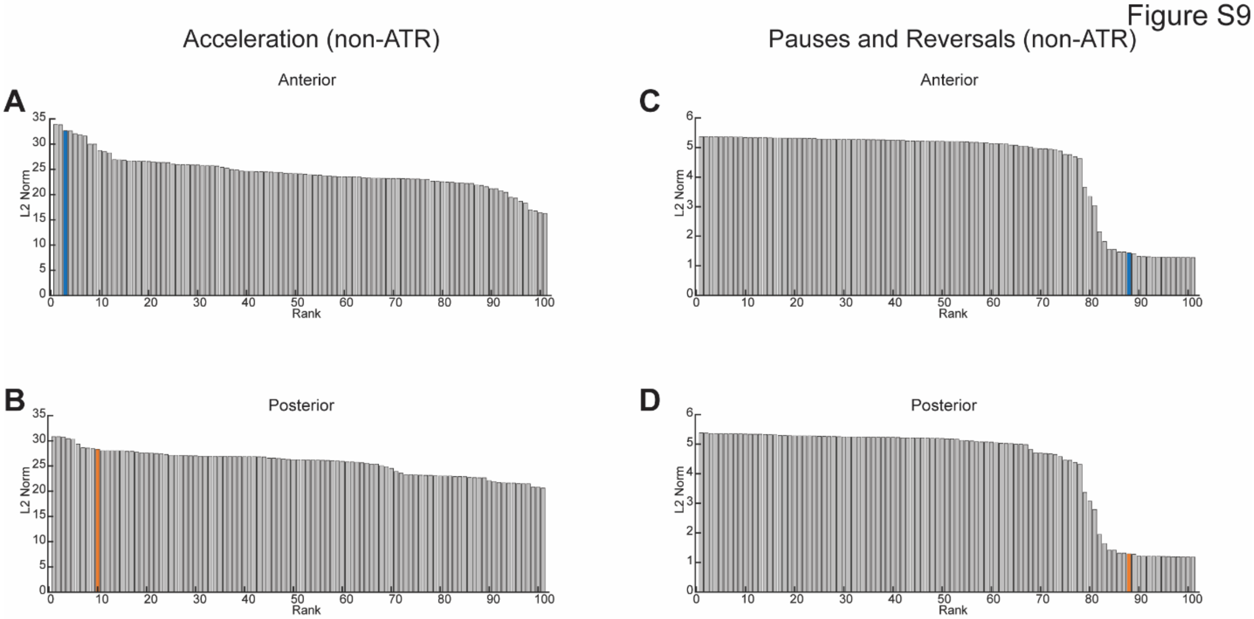

Figure S9: Significance test results of nonATR control data for linear filters for PVD. Results from shuffled data significance tests for linear filters computed for activation of PVD in control, or nonATR-fed animals, in Figure 3. (A,B) Significance test results for computed filters for acceleration for anterior (A) and posterior (B) segments of PVD. (C,D) Significance test results for computed filters for pauses and reversals for anterior (C) and posterior (D) segments of PVD. Bar plots represent the magnitude of filters, computed as the L2 norm, and are plotted in ranked order from highest to lowest magnitude. Colored bar represents appropriately computed filter, gray bars represent filters computers with shuffled data.

Figure S10

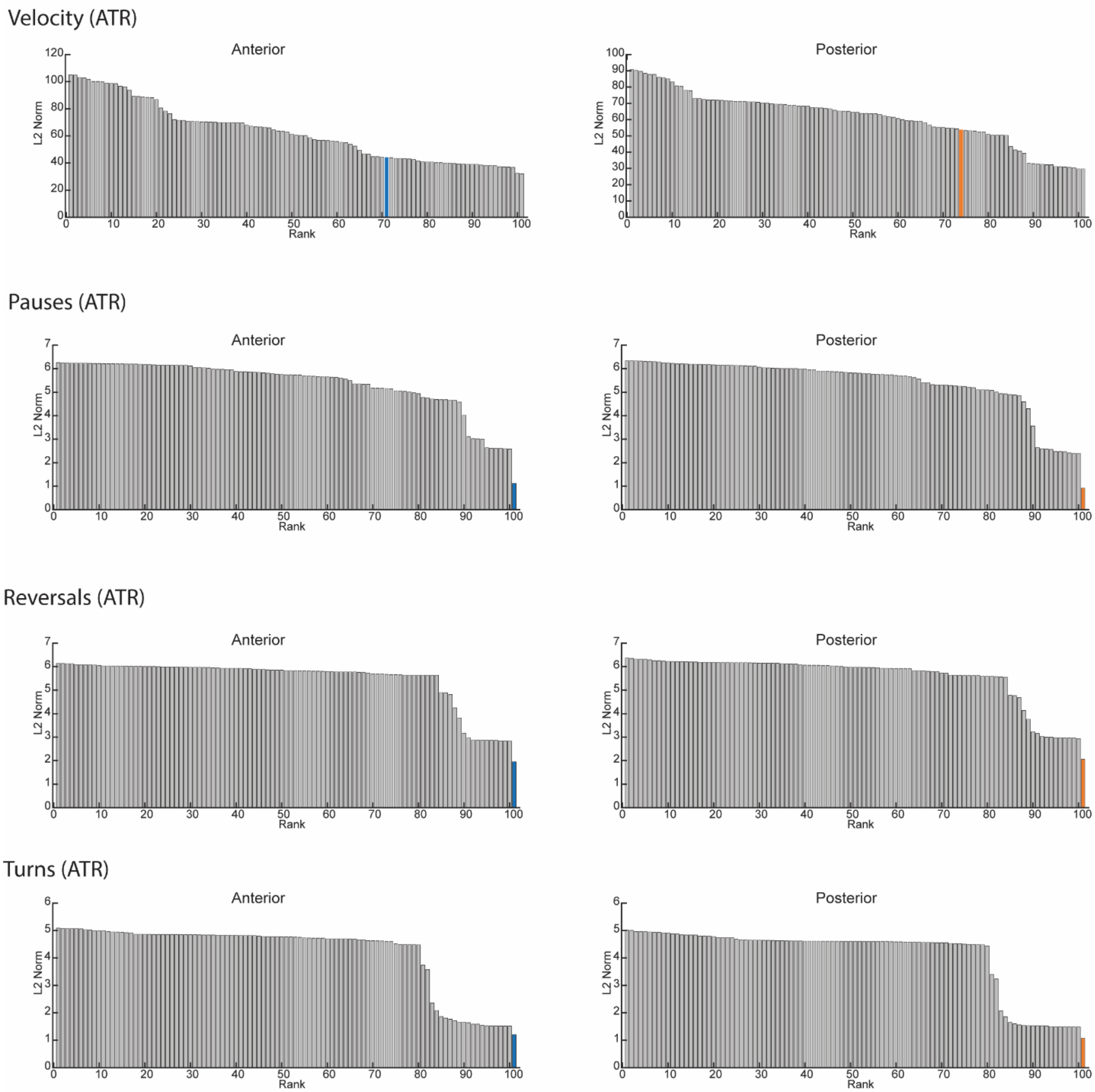

Figure S10: Significance test results for additional linear filters for PVD. Results from shuffled data significance tests for linear filters computed for activation of PVD in ATR-fed animals in Figure S7. Significance test results for computed filters for velocity, pauses, reversals, and turns, for anterior and posterior segments of PVD. Bar plots represent the magnitude of filters, computed as the L2 norm, and are plotted in ranked order from highest to lowest magnitude. Colored bar represents appropriately computed filter, gray bars represent filters computers with shuffled data.

Figure S11

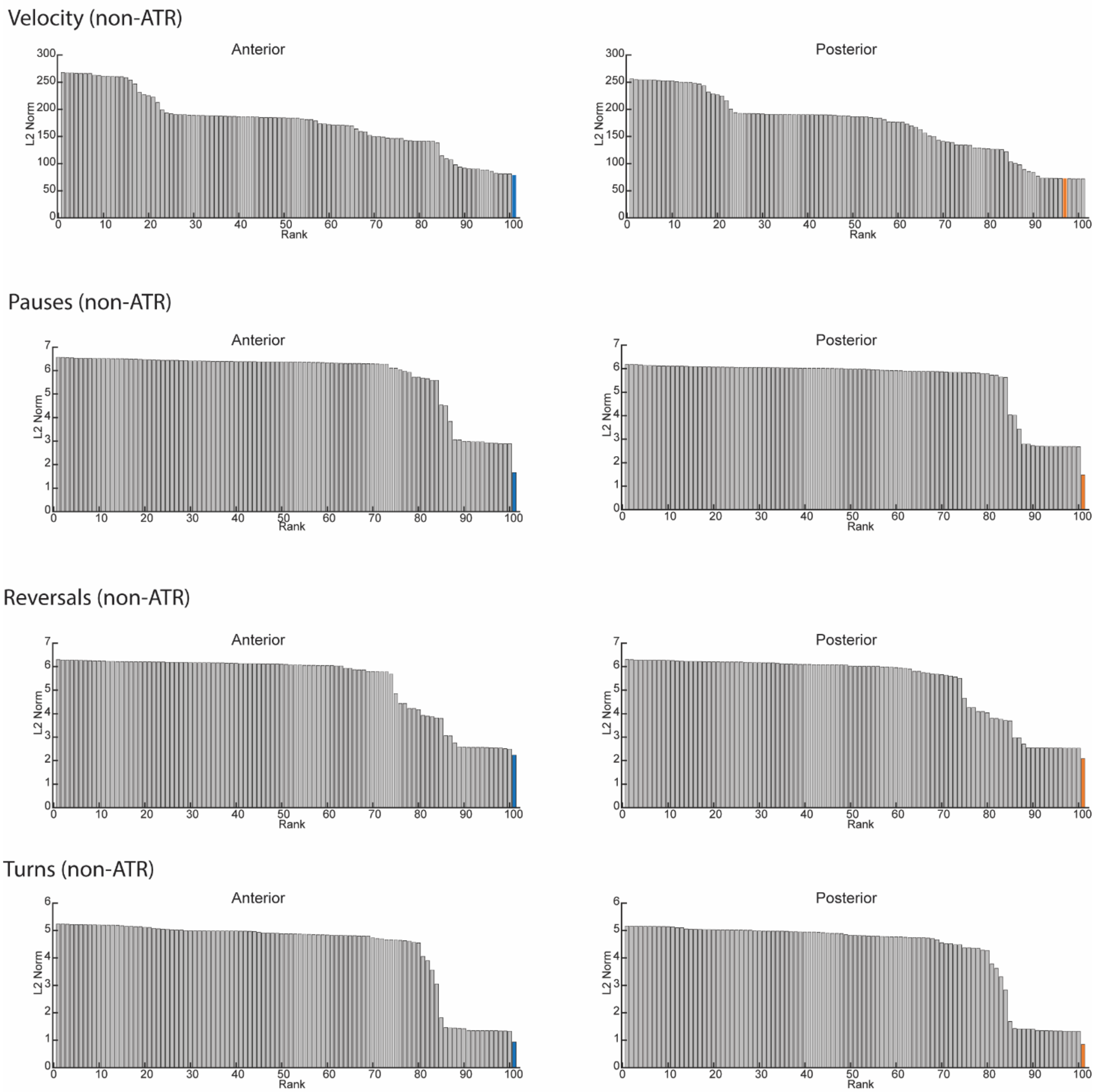

Figure S11: Significance test results of nonATR control data for additional linear filters for PVD. Results from shuffled data significance tests for linear filters computed for activation of PVD in control, or nonATR-fed animals, in Figure S7. Significance test results for computed filters for velocity, pauses, reversals, and turns, for anterior and posterior segments of PVD. Bar plots represent the magnitude of filters, computed as the L2 norm, and are plotted in ranked order from highest to lowest magnitude. Colored bar represents appropriately computed filter, gray bars represent filters computers with shuffled data.

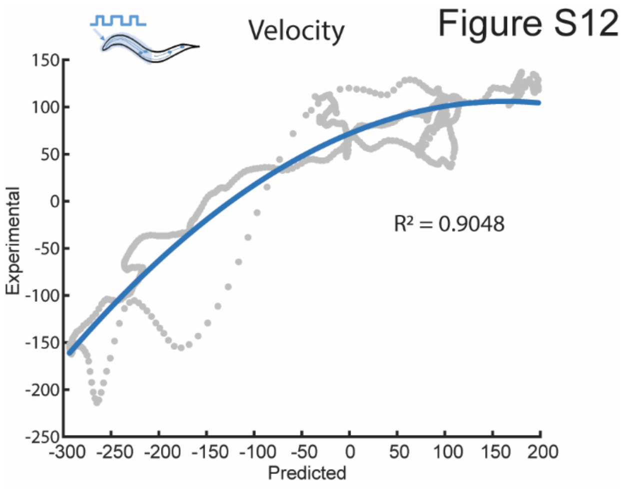

Figure S12: Static nonlinear filters for velocity. Static nonlinear filters fitted for predicted values from the linear filter (x-axis) against experimental values (y-axis) when stimulating the anterior TRNs. Linear filters and experimental values are subsets of data used in Figure 2 (n>1,730 for all conditions). Colored traces represent computed nonlinear filters and gray dots represent independent time-points from measured and predicted values.

Figure S13

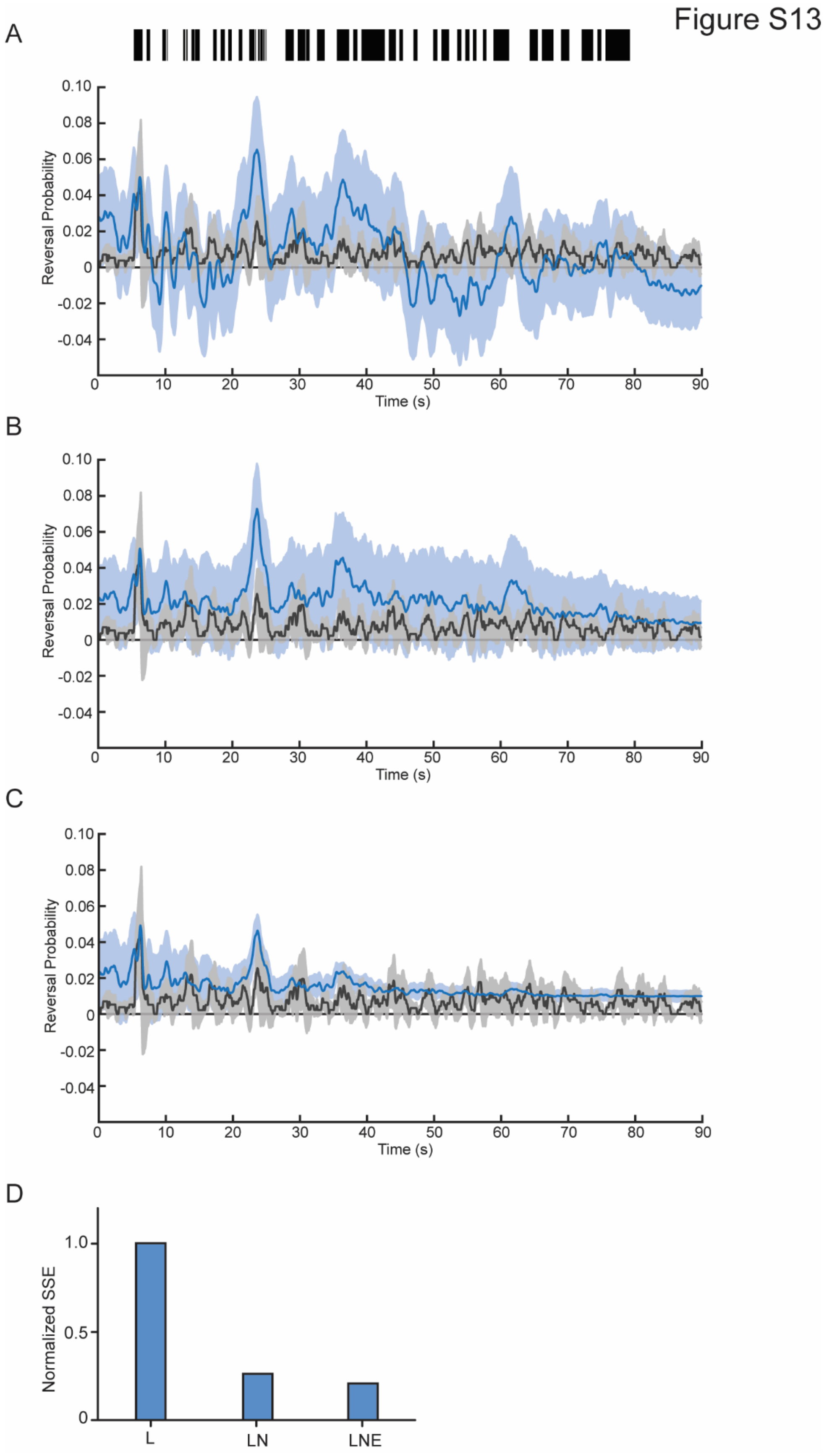

Figure S13: Model predictions of reversal initiations. Comparison of model predictions of reversal transitions (blue) and experimental traces (black) when using A) only the linear filter, B) a linear-nonlinear (LN) model, and C) an additional exponential component (LNE). For experimental data, dark line and shade represent average and SEM, respectively (n = 31 animals). For model predictions, dark line represents model prediction and shaded area represents the 95% confidence interval (Methods). Probability of reversal transitions is computed as the average of animals initiating a reversal at that time point. D) Comparison of performance of models, computed as the sum of squared error (SSE) and normalized to the linear model performance value.

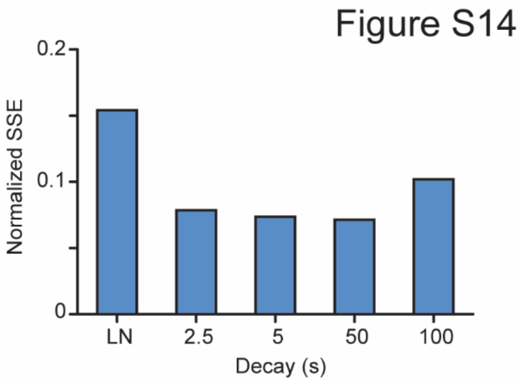

Figure S14: Comparison of decay factors. Comparison of model predictions of velocity for various exponential decay factors. Exponential decays of 2.5s, 5s, 50s, and 100s were tested, with 50s showing the best fit. Performance of models is computed as the sum of squared error (SSE), normalized to the linear model performance value.

Figure S15

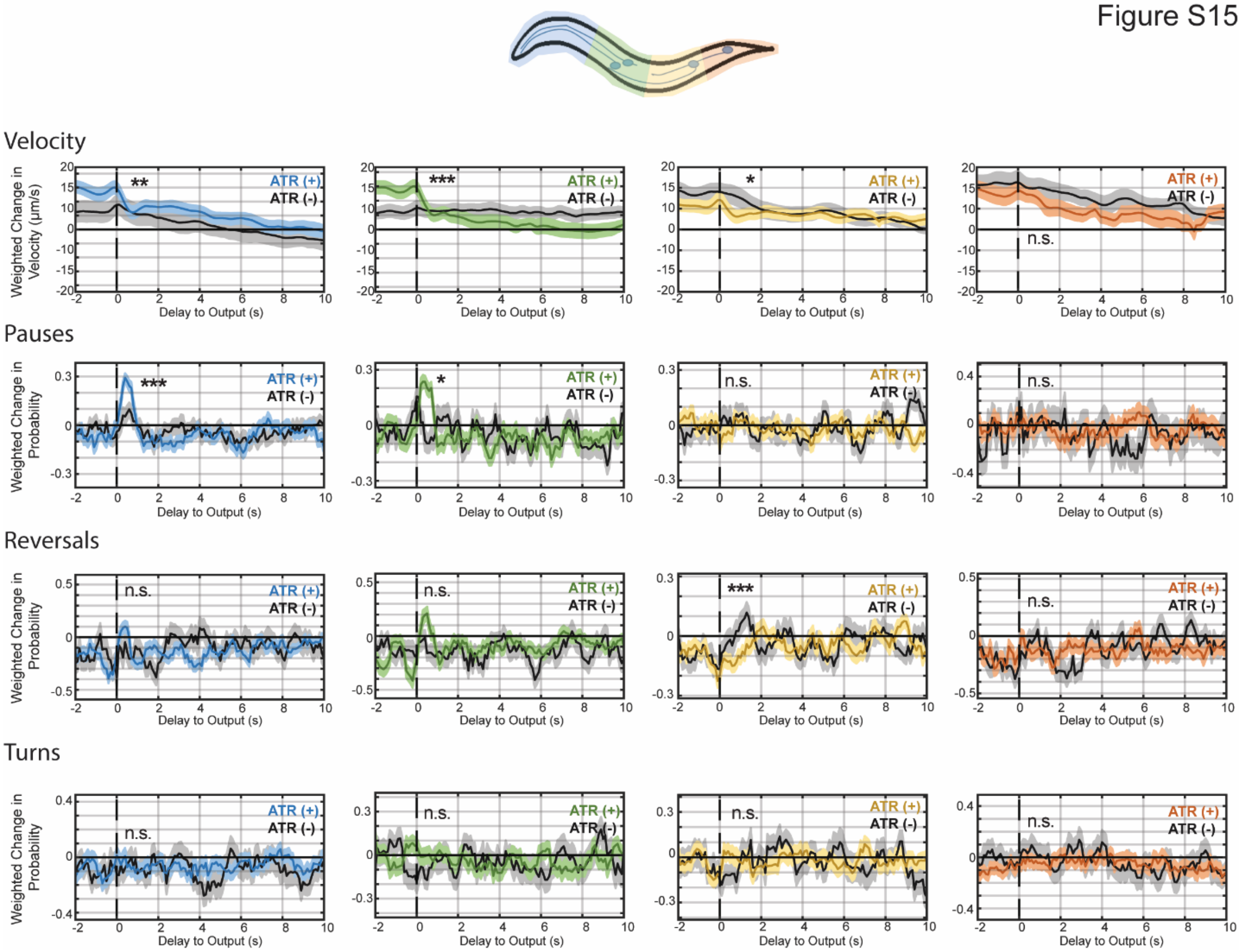

Figure S15: Additional filters for spatially refined analysis of TRNs Linear filters computed for various behaviors when stimulating the most anterior quarter (left), the second-most anterior quarter (second from left), the second-most posterior quarter (second from right), and the most posterior quarter (right) of the TRNs with an m-sequence signal. Dark line and light shade represent BWA and SEM, respectively. Colored plots represent filters computed from ATR-fed animals, black plots represent filters computed from control (not ATR-fed) animals. Statistical significance of filters determined by comparison with shuffled data (Fig S18-S19, ***Top 1%, ** Top 2%, * Top 3%, and not significant otherwise). Sample Sizes are listed Table S1.

Figure S16

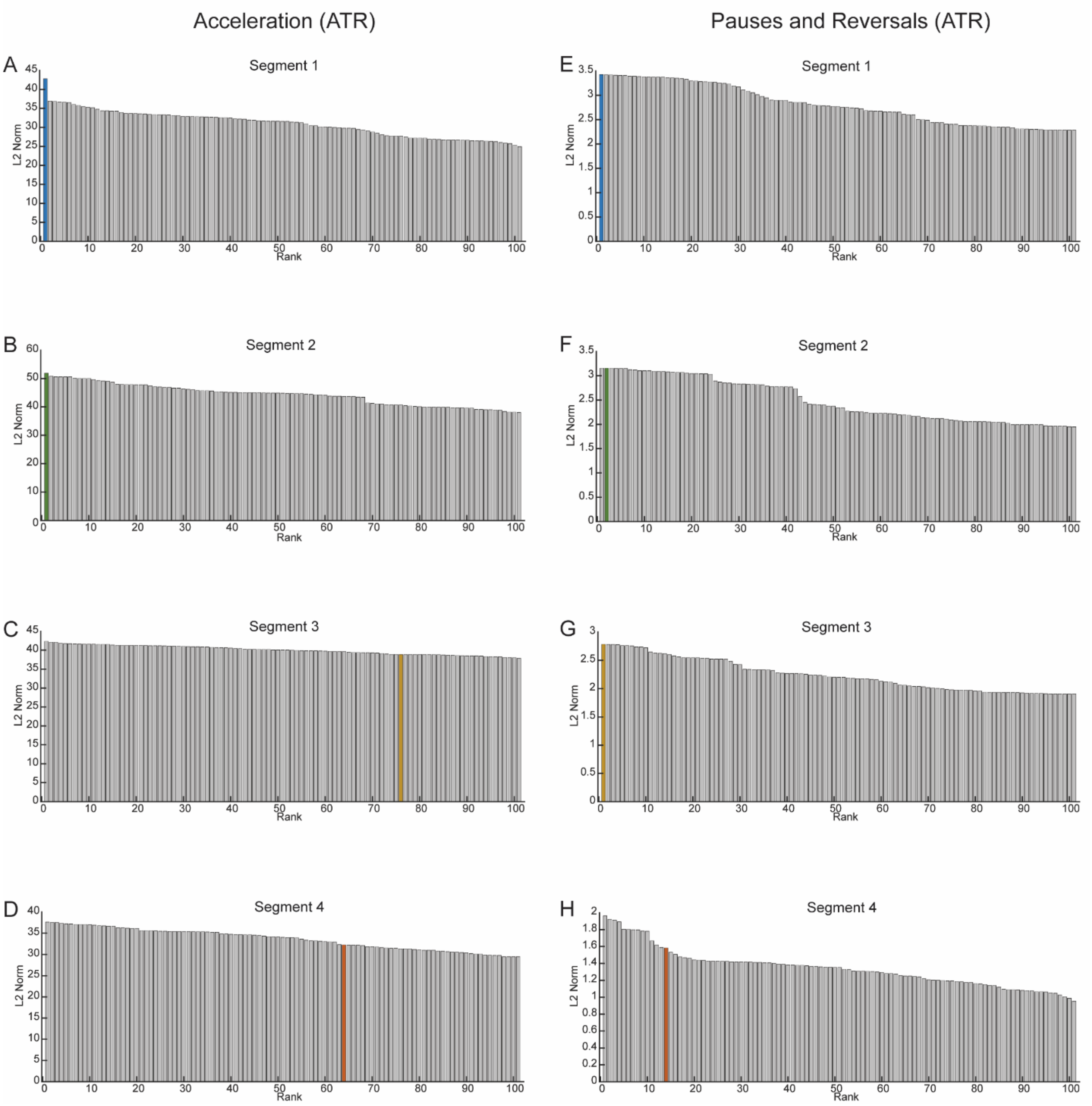

Figure S16: Significance test results for linear filters for spatially refined TRN analysis. Results from shuffled data significance tests for linear filters computed for activation of TRNs in ATR-fed animals in Figure 6. (A-D) Significance test results for computed filters for acceleration for most anterior (A), second-most anterior (B), second-most posterior (C), and most posterior (D) segments of TRNs. (E-H) Significance test results for computed filters for pauses and reversals for most anterior (E), second-most anterior (F), second-most posterior (G), and most posterior (H) segments of TRNs. Bar plots represent the magnitude of filters, computed as the L2 norm, and are plotted in ranked order from highest to lowest magnitude. Colored bar represents appropriately computed filter, gray bars represent filters computers with shuffled data.

Figure S17

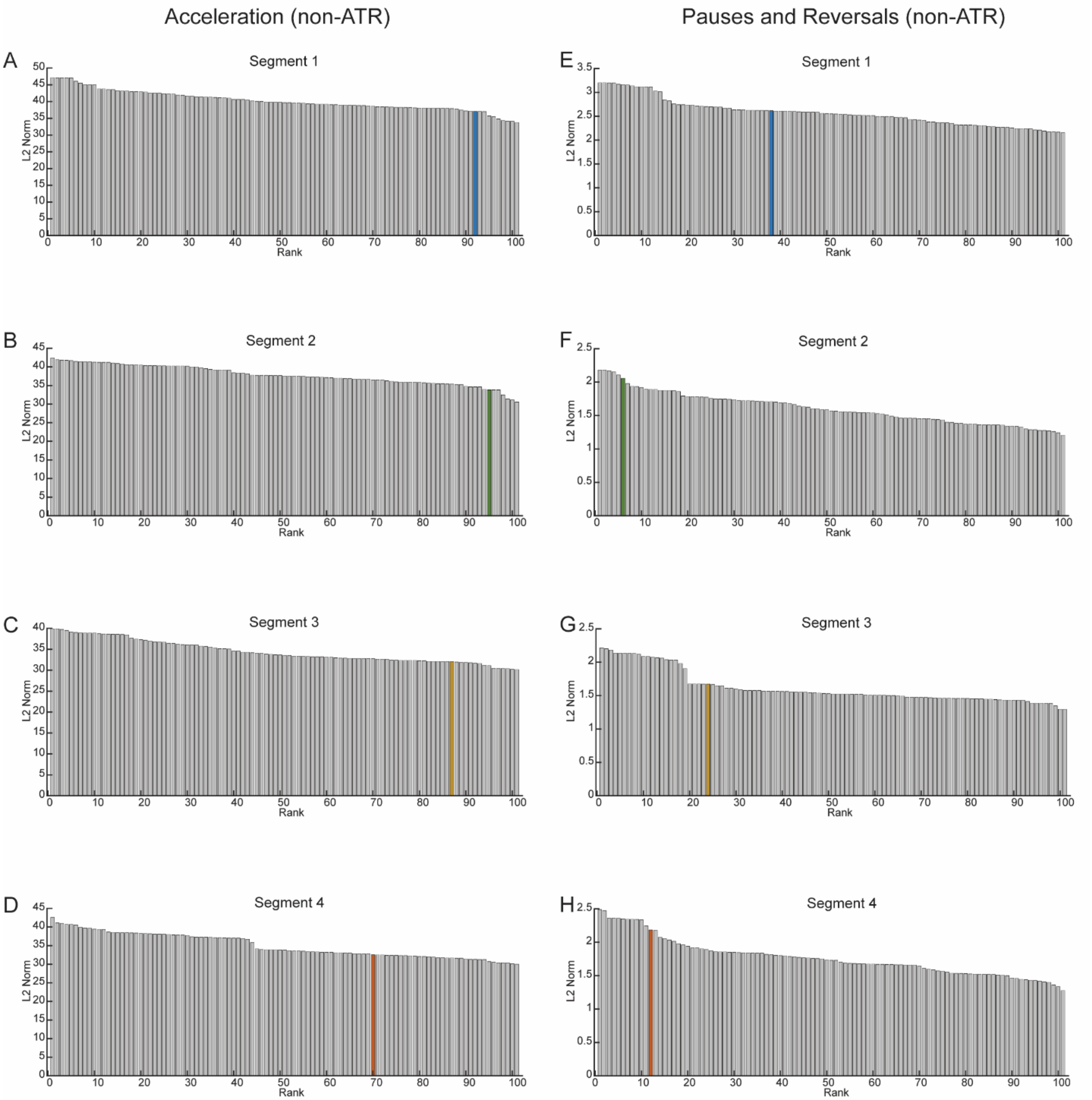

Figure S17: Significance test results of nonATR control data for linear filters for spatially refined TRN analysis. Results from shuffled data significance tests for linear filters computed for activation of TRNs in control, or nonATR-fed animals, in Figure 6. (A-D) Significance test results for computed filters for acceleration for most anterior (A), second-most anterior (B), second-most posterior (C), and most posterior (D) segments of TRNs. (E-H) Significance test results for computed filters for pauses and reversals for most anterior (E), second-most anterior (F), second-most posterior (G), and most posterior (H) segments of TRNs. Bar plots represent the magnitude of filters, computed as the L2 norm, and are plotted in ranked order from highest to lowest magnitude. Colored bar represents appropriately computed filter, gray bars represent filters computers with shuffled data.

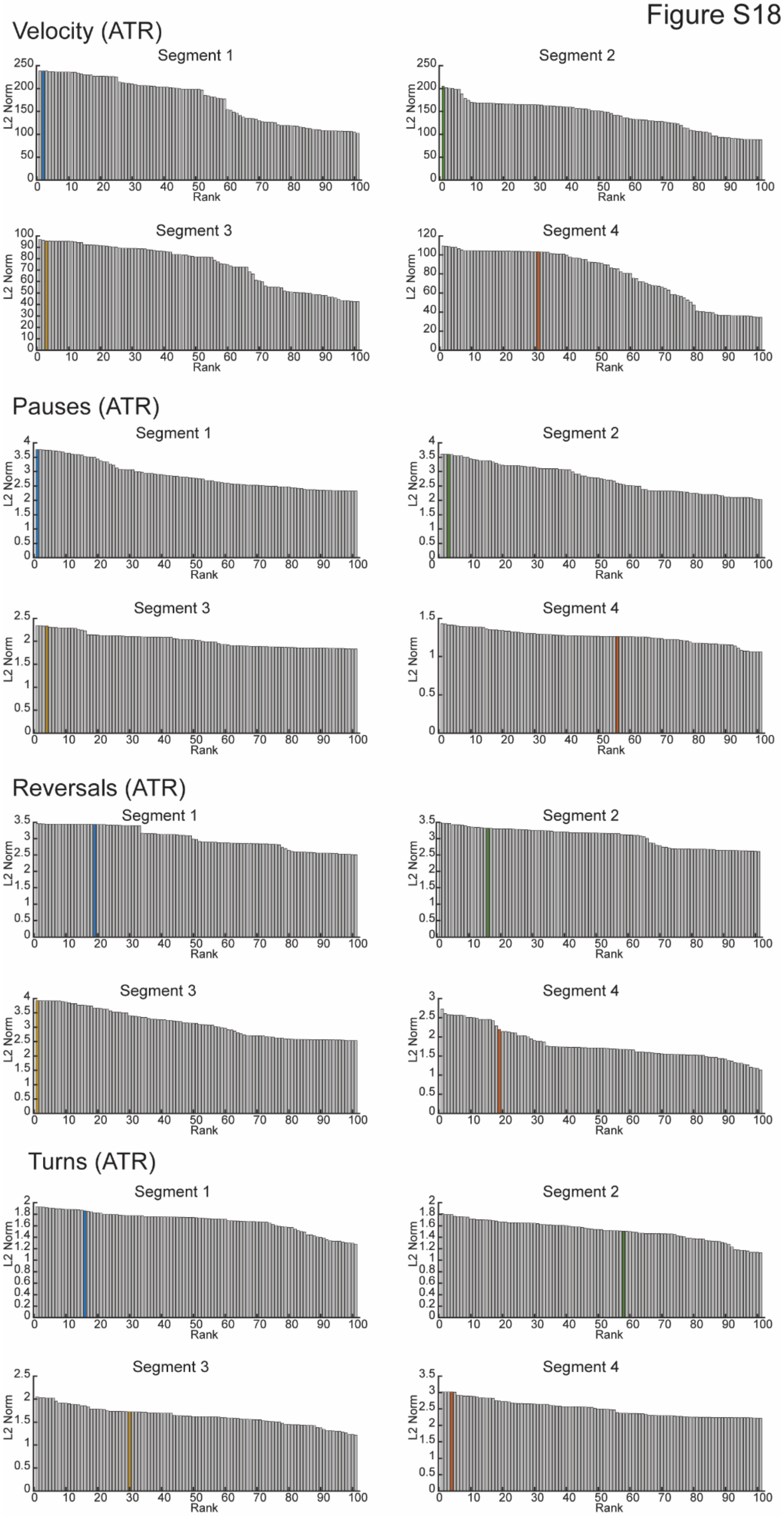
Velocity (ATR)
Segment 1
Segment 2
Segment 3
Segment 4
Pauses (ATR)
Segment 1
Segment 2
Segment 3
Segment 4
Reversals (ATR)
Segment 1
Segment 2
Segment 3
Segment 4
Turns (ATR)
Segment 1
Segment 2
Segment 3
Segment 4
L2 Norm
Rank

Figure S18

Figure S18: Significance test results for additional linear filters for spatially refined TRN analysis. Results from shuffled data significance tests for linear filters computed for activation of TRNs in ATR-fed animals in Figure S15. Significance test results for computed filters for velocity, pauses, reversals, and turns for most anterior, second-most anterior, second-most posterior, and most posterior segments of TRNs. Bar plots represent the magnitude of filters, computed as the L2 norm, and are plotted in ranked order from highest to lowest magnitude. Colored bar represents appropriately computed filter, gray bars represent filters computers with shuffled data.

Figure S19

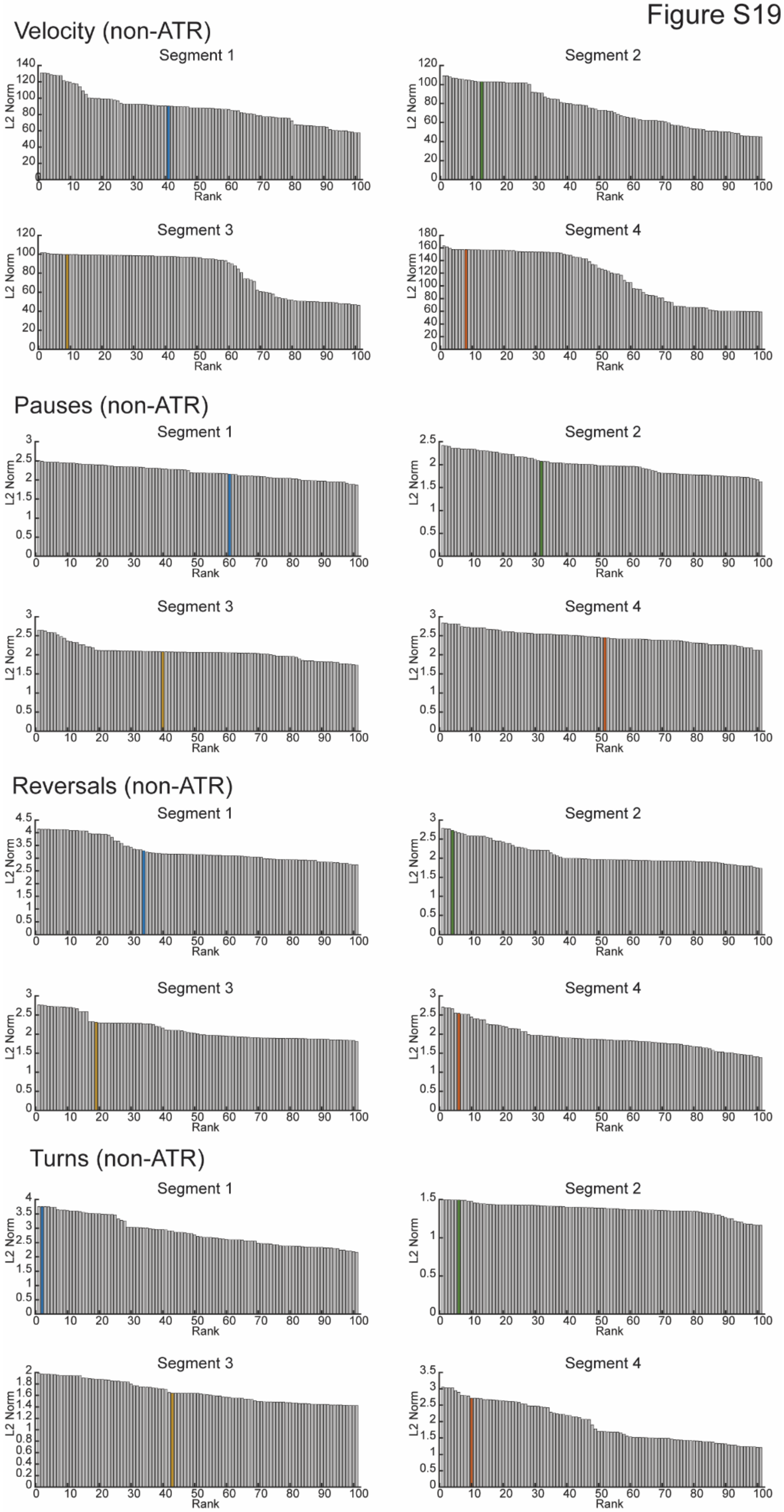
Velocity (non-ATR)
Segment 1
Segment 2
Segment 3
Segment 4
Pauses (non-ATR)
Segment 1
Segment 2
Segment 3
Segment 4
Reversals (non-ATR)
Segment 1
Segment 2
Segment 3
Segment 4
Turns (non-ATR)
Segment 1
Segment 2
Segment 3
Segment 4
L2 Norm
Rank

Figure S19: Significance test results of nonATR control data for additional linear filters for spatially refined TRN analysis. Results from shuffled data significance tests for linear filters computed for activation of TRNs in control, or nonATR-fed animals, in Figure S15. Significance test results for computed filters for velocity, pauses, reversals, and turns for most anterior, second-most anterior, second-most posterior, and most posterior segments of TRNs. Bar plots represent the magnitude of filters, computed as the L2 norm, and are plotted in ranked order from highest to lowest magnitude. Colored bar represents appropriately computed filter, gray bars represent filters computers with shuffled data.

Figure S20

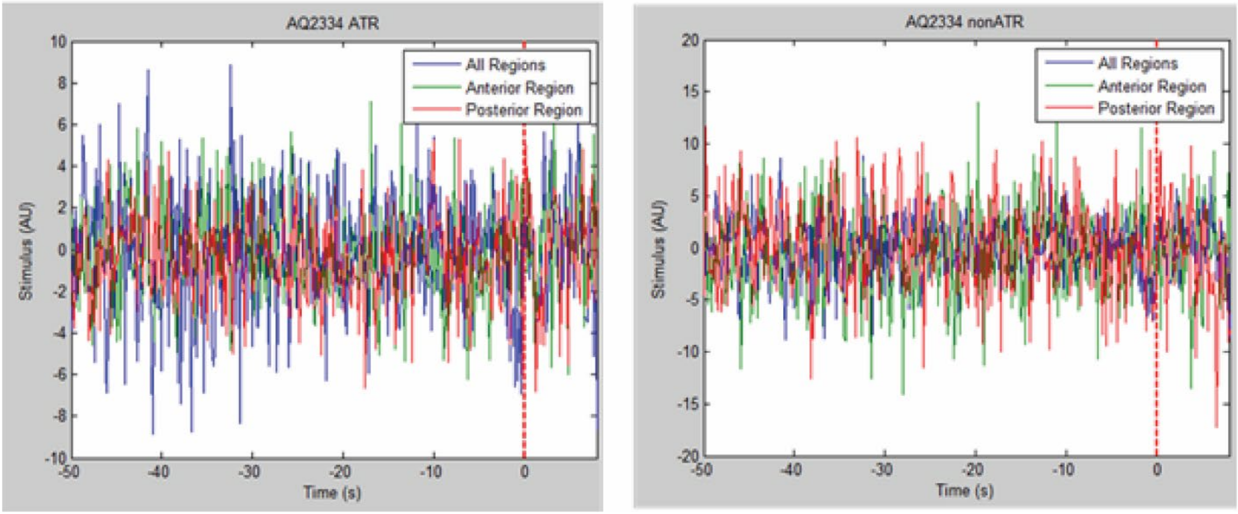

Figure S20: Sample negative results of computed filters using a different white noise signal. Linear filters computed for acceleration in response to activation of anterior TRNs, posterior TRNs, or both, from previous experimental trials. Lack of significant peaks and presence of noise illustrates that this signal did not induce reliable responses, and this signal cannot be used for this type of analysis. In this example, the signal was a Gaussian white noise signal, where the light intensity was modulated as a Guassian white noise variable, and values were modulated with a frequency of 10Hz.

Figure	Segment	Behavior	ATR		nonATR	
			Time-Points or Events	Animals	Time-Points or Events	Animals
Figure 1D	Anterior	Acceleration	88031	113	75618	98
Figure 2	Anterior (1)	Acceleration	88031	113	75618	98
		Pauses + Reversals	1766	113	1715	98
	Anterior (2)	Acceleration	36597	47	38238	50
		Pauses + Reversals	492	47	422	50
	Posterior	Acceleration	56744	61	47281	49
		Pauses + Reversals	667	61	571	49
Figure S1	Anterior (1)	Velocity	88025	113	75618	98
		Pauses	643	113	1026	98
		Reversals	549	113	163	98
		Turns	745	113	876	98
	Anterior (2)	Velocity	36606	47	38238	50
		Pauses	156	47	218	50
		Reversals	203	47	37	50
		Turns	176	47	242	50
	Posterior	Velocity	56742	61	47281	49
		Pauses	197	61	191	49
		Reversals	212	61	133	49
		Turns	280	61	272	49
Figure 3	Anterior	Acceleration (max)	53878	61	45479	51
		Acceleration (min)	53816	61	45404	51
		Pauses + Reversals	491	61	555	51
	Posterior	Acceleration (max)	53878	61	45479	51
		Acceleration (min)	53807	61	45464	51
		Pauses + Reversals	491	61	555	51
Figure S4	Anterior	Velocity	53834	61	45474	51
		Pauses	98	61	62	51
		Reversals	94	61	161	51
		Turns	313	61	340	51
	Posterior	Velocity	53834	61	45474	51
		Pauses	98	61	62	51
		Reversals	94	61	161	51
		Turns	313	61	340	51
Figure 4	Anterior	Acceleration	600	113		
Figure S5		Velocity	600	113		
		Pauses + Reversals	600	113		
Figure 6	Seg1	Acceleration	58713	67	35477	40
		Pauses + Reversals	538	67	302	40

	Seg2	Acceleration	59683	69	30729	35
		Pauses + Reversals	434	69	160	35
	Seg3	Acceleration	56490	66	28875	33
		Pauses + Reversals	366	66	127	33
	Seg4	Acceleration	62815	71	32219	36
		Pauses + Reversals	545	71	194	36
Figure S9	Seg1	Velocity	58662	67	35502	40
		Pauses	129	67	110	40
		Reversals	409	67	192	40
		Turns	272	67	134	40
	Seg2	Velocity	59650	69	30729	35
		Pauses	100	69	65	35
		Reversals	334	69	95	35
		Turns	323	69	165	35
	Seg3	Velocity	56480	66	28876	33
		Pauses	149	66	50	33
		Reversals	217	66	77	33
		Turns	251	66	112	33
	Seg4	Velocity	62808	71	32216	36
		Pauses	224	71	58	36
		Reversals	321	71	136	36
		Turns	342	71	145	36

Table S1: Sample sizes for computed linear filters in figures 1, 2, S1, 3, S4, 4, S5, 6, and S9.
